# Pancreatic cancer and FOLFIRINOX: a new era and new questions

**DOI:** 10.1002/cam4.433

**Published:** 2015-02-19

**Authors:** Robert De W Marsh, Mark S Talamonti, Matthew Harold Katz, Joseph M Herman

**Affiliations:** 1 Department of Medicine, NorthShore University HealthSystemEvanston, Illinois, 60201; 2 Department of Surgery, NorthShore University HealthSystemEvanston, Illinois, 60201; 3 Department of Surgical Oncology, The University of Texas MD Anderson Cancer CenterHouston, Texas; 4 Department of Radiation Oncology and Molecular Radiation Sciences, Sidney Kimmel Comprehensive Cancer Center, Johns Hopkins HospitalBaltimore, Maryland

**Keywords:** Chemotherapy, FOLFIRINOX, genomics, modifications, pancreatic cancer, toxicity

## Abstract

FOLFIRINOX (FFX) was introduced to clinical practice in 2010 following publication of the PRODIGE 4/ACCORD 11 study, which compared this novel regimen to gemcitabine in metastatic pancreatic cancer. Median overall survival, progression-free survival, and objective responses were all superior with FFX and there was improved time to definitive deterioration in quality of life. Despite initial concerns over toxicity, there has been rapid uptake of this regimen, both revolutionizing management and opening the door to innovative research. As experience with FFX has accrued, many questions have arisen including the management of toxicities, the impact of frequent modifications, the optimal number of cycles, integration with other regimens and modalities, interpretation of radiologic and serologic response, utility of molecular signatures, and potential benefit in unique clinical settings such as pre- and postsurgery. This review will closely examine these issues, not only to summarize current knowledge but also to fuel scientific debate.

## Introduction

### Historical context

Previously published studies have suggested that combination therapy could be an improvement on gemcitabine alone. These include the phase III study of gemcitabine versus gemcitabine plus erlotinib 1, the phase III study of gemcitabine versus gemcitabine plus capecitabine 2, and the phase II study of GTX (gemcitabine, taxotere, and capecitabine) 3,4. In the first study, overall survival (OS) (median 6.24 vs. 5.91 months, HR = 0.82, 95% CI = 0.69–0.99; *P* = 0.038), 1-year survival (23% vs. 17%; *P* = 0.023), and progression-free survival (HR = 0.77, 95% CI = 0.64–0.92; *P* = 0.004) were better with gemcitabine plus erlotinib. In the second study, objective response rate (19.1% vs. 12.4%; *P* = 0.034) and progression-free survival (HR = 0.78; 95% CI = 0.66–0.93; *P* = 0.004) favored the combination and there was a trend toward improved OS (7.1 vs. 6.2 months, HR = 0.86, 95% CI = 0.72–1.02; *P* = 0.08). In the GTX study, median progression-free survival of responders was 6.3 months (95% CI = 4.4–10.4 months) and median survival was 11.2 months (95% CI = 8.1–15.1 months). While certainly of interest, the clinical benefit of these regimens was either marginal, of uncertain impact on quality of life, or achieved in very small numbers, resulting in sporadic and unenthusiastic uptake.

### FOLFIRINOX

Promising phase II results with FOLFIRINOX (FFX) 5 (oxaliplatin 85 mg/m^2^, leucovorin 400 mg/m^2^, irinotecan 180 mg/m^2^, bolus 5-fluorouracil 5FU) ( 400 mg/m^2^, infusional 5FU 2400 mg/m^2^ over 46 h, every 14 days) were confirmed in a sentinel phase III study (PRODIGE 4/ACCORD 11) 6, which randomized patients ≤75 years of age with metastatic pancreatic cancer and an ECOG PS of 0 or 1, to receive either gemcitabine or FFX. With a median follow-up of 26.6 months and with 171 patients in each arm (38% of patients had lesions in the head of the pancreas with 14.3% requiring biliary stents), the median survival with FFX was 11.1 months versus 6.8 months for gemcitabine (*P* < 0.001, HR = 0.57, 95% CI = 0.45–0.73). More impressively, 1-year survival was 48.4% versus 20.6%, respectively, and this difference was sustained at 18 months, 18.6% versus 6%. Quality of life measures, equally, strongly favored the FFX group 7.

While toxicity was not inconsequential (45.7% grade 3 or 4 neutropenia, 5.4% febrile neutropenia, 12.7% diarrhea, 9.1% thrombocytopenia, 9.0% sensory neuropathy), oncologists rapidly adopted the FFX regimen following the 2010 ASCO meeting 8. Many questions have now arisen such as: best management of common and uncommon toxicities; potential impact of adjustments to the original regimen; number of cycles administered for optimal results; innovative strategies in early disease; radiologic and serologic assessment of response; evolving data on integration into overall treatment planning; and utility of molecular profiling.

In order to derive the data used in this review, all relevant papers in Medline, CANCERLIT, and Index Medicus together with meeting abstracts from ASCO, ASTRO, and AACR since 1990, were examined. No ethnic or racial group or gender was excluded. Approximately 65% of discovered references have been included based on relevance, timeliness, and quality of data.

## How is Toxicity of FFX Best Managed?

As with usual practice, reduction in individual drug dosing is a standard approach for many of the common complications such as low blood counts, fever, infection, diarrhea, weight loss, and fatigue. However, some problems engendered by FFX are either idiosyncratic, not dose related, or not manageable with simple dose reduction and may require more innovative strategies (Table 1).

**Table 1:** Management of FOLFIRINOX toxicity

Toxicity	Strategy	Concern
Low blood counts, fatigue, diarrhea, mucositis	Decrease doses of one or more of the drugs; lomotil/pegfilgrastim	Decreased efficacy of therapy; bone pain
Low platelet counts despite appropriate dose reduction	Splenectomy—surgical or via interventional radiology	Pain; abscess formation; treatment delay
Acute allergic reaction to oxaliplatin infusion	Desensitization protocol and possible discontinuation	Ineffective to resolve problem; resources
Hypercholinergic reaction with cramping and sweating	Slow infusion rate and premedicate with atropine	Prolonged treatment time; resources
Oral dysesthesia with sense of swollen tongue	Slow infusion rate and warm drink	Prolonged treatment time; anxiety; resources
Weakness, paralysis, and even coma	Maintenance of normal potassium and calcium prior to and during infusion	Patient anxiety; staff anxiety; imperfect results

If platelet counts are problematic despite dose modifications, then splenectomy, either surgical 9 or by endovascular means using an embolic approach 10, can help in selected patients. The typical phenotype would be someone who is responding to chemotherapy, with a good functional status, but who has isolated thrombocytopenia (<90 × 10^3^/*μ*L). In the surgical series, counts increased significantly (*P* < 0.01) with a mean value of 87 × 10^3^/*μ*L prior to treatment and 425 × 10^3^/*μ*L on discharge (average 3 days later). All patients were able to resume chemotherapy within a median of 11.5 days (range 6–27). The IR procedure could be particularly useful in those either too frail for surgery or for whom surgery is relatively contraindicated (e.g., disease in the splenic hilum or carcinomatosis). Complications of postoperative pain and splenic abscess are limiting factors 11 and relative efficacy is unknown.

Infusion reactions are common and desensitizing protocols may be needed 12. A significant hypercholinergic response with excess salivation, cramping, and sweating, related to the piperidine structure of irinotecan, which mimics a cholinergic drug when metabolized by esterases to form SN-38, is not unusual 13. The potentiating role of oxaliplatin is real but not well understood 14. Slowing of the infusion, aggressive medication with atropine, and a proton pump inhibitor may be required.

The common problems of oral dysesthesia and thick tongue, and the rare complication of total body weakness, near paralysis and even coma from oxaliplatin may be difficult to manage. Slowing the infusion and a warm drink works best for the former, while aggressive correction of serum potassium and calcium prior to, and following, the infusion may resolve the latter 15,16.

## Do Modifications to the FFX Regimen Matter?

Oncologists in the United States and elsewhere were anxious to use FFX, but initially concerned about toxicity, particularly in patients with lesions in the head of the pancreas and with biliary stents. A Canadian report suggested that there could be considerable toxicity when the regimen is used outside of a clinical study and in community centers 17. In their series of 46 patients, there were 3 (7%) treatment-related deaths, 54% of patients were hospitalized with sepsis, 33% had neutropenia grade ≥3, 15% had diarrhea grade ≥3, and 4 (9%) patients had febrile neutropenia.

With this scenario in mind, many modifications have been made (Table 2). Initially, physicians removed the bolus of 5FU, which is notably myelosuppressive, with some adding pegfilgrastim 6 mg on day 3 or 4. Commonly referred to as “mFOLFIRINOX,” this seems to be the way it is often used today 18. Historically, a bolus of 5FU has been used in the majority of fluoropyrimidine regimens, together with a more prolonged infusion to maximize total exposure 19. A Japanese study shows that the bolus contributes significantly to the overall exposure to 5FU via AUC 20. In addition, 5FU functions differently depending on how it is administered 21 and thus, theoretically, the omission of the bolus could lead to loss of efficacy. Data reported at the 2014 GI ASCO meeting suggest, however, that this may not be the case, and longer follow-up will be needed for clarification 22.

**Table 2:** FOLFIRINOX dose modifications and results

Author	Modification	Results/comments
Mahaseth et al. 18	Drop 5FU bolus	Grade 4 neutropenia 3%
	Add pegfilgrastim 6 mg	Grade 3/4 diarrhea 13%, fatigue 13%
		OS 9.0 months, PFS 8.5 months
Blazer et al. 24	Drop 5FU bolus	Add pegfilgrastim 6 mg
	Decrease irinotecan to 165 mg/m^2^	Grade 3/4 neutropenia or thrombocytopenia 0%
	46% further dose reductions for other toxicities	
Gunturu et al. 25	Median dose intensity 5FU bolus 57%	Grade 3/4 neutropenia 6.4%
	Median dose intensity oxaliplatin 88%	Grade 3/4 fatigue 9.6%
	Median dose intensity irinotecan 64%	CR plus PR 31.6%
Metges et al. 27	Median dose intensity 5FU bolus 82%	Grade 3/4 hematologic and neurotoxicity 32%
	Median dose intensity oxaliplatin 78%	Response rate 39%
	Median dose intensity irinotecan 81%	PFS 6.5 months
		OS 10.9 months
Alessandretti et al. 26	Drop 5FU bolus	Grade 3/4 neutropenia 21% or thrombocytopenia 5%
	Decrease 5FU infusion to 2000 mg/m^2^	Grade 3/4 fatigue 15.7%
	Decrease oxaliplatin to 50 mg/m^2^	CR plus PR 31.7%
	Decrease irinotecan to 135 mg/m^2^	OS and PFS not reached at 4 months
	Add pegfilgrastim 6 mg	
James et al. 22	Decrease 5FU bolus 25%	Grade 3/4 neutropenia 17% or thrombocytopenia 11.3%
	Decrease irinotecan 25%	Grade 3/4 fatigue 11.3%
	Add pegfilgrastim 6 mg	CR plus PR 29%

A further dilemma concerns the omission of leucovorin, should the bolus of 5FU be removed. Previous dose-finding studies of infusional 5FU with leucovorin clearly demonstrated that there is considerable synergy, and that omission of leucovorin results in less toxicity 23, suggesting that efficacy could equally be impacted. Absent real data, and given the low cost of leucovorin, it seems reasonable to leave it untouched.

Ohio State physicians reported their experience with limiting irinotecan to 165 mg/m^2^ in addition to these changes, in either locally advanced or borderline resectable disease. They concluded that the modified regimen was effective and well tolerated with no episodes of grade 3 or 4 neutropenia/thrombocytopenia, but with 46% of patients requiring a dose reduction for other toxicities 24. Similarly, physicians at Yale reported that in their hands dose reductions were common (relative dose intensities: oxaliplatin 88%, irinotecan 64%, bolus 5FU 57%, infusional 5FU 100%, compared to oxaliplatin 78%, irinotecan 81%, and 5FU 82%—PRODIGE 4/ACCORD 11) 25. Despite these modifications, efficacy was comparable to that of the original regimen—response (CR + PR 33%—similar to historical data 31.6%; *P* = 0.21), and toxicity was notably less (grade 3 or 4 neutropenia 6.4%, *P* < 0.0001; fatigue 9.6%, *P* < 0.02).

For frail and elderly patients, additional adjustments have been made. In a series of 19 patients over age 65, the bolus of 5FU was dropped and doses of both oxaliplatin and irinotecan were lowered (5FU 2000 mg/m^2^ over 46 h, oxaliplatin 50 mg/m^2^, irinotecan 135 mg/m^2^) 26. Grade 3/4 toxicities were reported in 10 patients: nausea/vomiting in one, diarrhea in one, fatigue in three, neutropenia in four, thrombocytopenia in one, and febrile neutropenia in three—all manageable. A follow-up study by the original investigators in the PRODIGE 4/ACCORD 11 study, based on their established criteria, showed that 81% of 242 patients required a dose reduction, but that this did not affect results (response rate 39% vs. 32%, PFS 6.5 vs. 6.4 months and OS 10.9 vs. 11.1 months) 27.

A biologically based refinement, using genotype-derived dosing of irinotecan via UGT1A1, the enzyme that inactivates SN-38 (the active metabolite of irinotecan) showed that those with a *28*28 genotype are at highest risk of severe neutropenia, *1*28 at intermediate risk, and *1*1 at lowest risk 28. Initial doses of irinotecan could be adjusted accordingly.

A close examination of clinicaltrials.gov confirms that the majority of regimens presently under investigation incorporate some modification of FFX.

## How is the Number of Treatment Cycles with FFX Determined?

The optimal number of treatment cycles is not well understood, but the goal of therapy (i.e., curative vs. palliative) is critical in this regard. The disease should be unambiguously defined as either resectable, borderline resectable, locally advanced unresectable, or metastatic. This has implications for ensuring that treatment is not unnecessarily modified, or conversely, that excessive treatment (and toxicity) is not given. This is simplest in a palliative setting, where duration and intensity of treatment is determined by response and quality of life. The median number of cycles in the original PRODIGE 4/ACCORD 11 study was 10, with a range of 1–47 6. In locally advanced and borderline resectable disease, it is common to use four cycles of FFX (± chemo/RT) in a neoadjuvant strategy (e.g., ALLIANCE/Intergroup study A021101). This is based on very limited data, and an alternative approach might be to treat to maximal response and/or maximum-tolerated dose. A retrospective study of this strategy in borderline (60%) and locally advanced, unresectable (40%) disease examined outcomes in 18 patients 29. An R0 resection was ultimately possible in 44% of patients, with a median number of six cycles (range 5–17) prior to surgery. A report on FFX plus chemo/RT in 22 patients with locally advanced, unresectable disease, examined use of an initial four cycles with an additional four cycles prior to chemo/RT, if disease was either stable or improved 30. A median of eight cycles was administered, with 12 patients taken to the OR and 5 (42%) were able to have an R0 resection. However, three patients developed distant recurrence within 81 days, confirming their dismal prognosis.

Steatohepatitis (irinotecan) and sinusoidal obstructive syndrome (oxaliplatin) are dose-related complications which effect outcome in liver resection for colorectal cancer 31. A Whipple operation, in and of itself, leads to an increase in hepatic steatosis 32. Further, a BMI exceeding 25 kg/m^2^, diabetes mellitus, and preexisting steatosis all significantly increase the risk of steatohepatitis and postoperative morbidity 33. These data suggest that the number of cycles be limited to the minimum necessary, as the effects on patients undergoing a Whipple operation are as yet unknown.

Complicating matters further, pancreatic cancer is clearly a heterogeneous disease 34. Aggressive subsets (if they do respond) may require three or four cycles of therapy before showing a decline in CA 19-9, implying response, and may conceivably require further cycles of chemotherapy prior to surgery.

In locally advanced (arterial encasement) or metastatic disease, initial intensive therapy could be followed by omission of either oxaliplatin or irinotecan (depending on which is more problematic) for continuation of a “maintenance program,” as this is strictly palliative therapy. While there are few publications on the efficacy of FOLFOX or FOLFIRI, those that do exist are positive 35–37.

## Is Preoperative or Postoperative FFX the Optimal Strategy for Potentially Resectable Disease?

One of the most intriguing questions currently under study is whether FFX will improve on results in the adjuvant therapy of resectable pancreatic cancer. A recent update of the CONKO-001 study shows that median OS is 22.8 months in the gemcitabine group versus 20.2 months in the observation group (HR = 0.76, *P* = 0.01) 38. OS at 5 and 10 years is 20.7% versus 12.2% and 10.4% versus 7.7%, respectively—all dismal numbers.

Studies comparing gemcitabine with combination therapy, and even vaccine therapy, have failed to improve on these results 39–41. There are no data as yet on FFX in the adjuvant setting (PRODIGE 24/ACCORD 24—gemcitabine vs. mFFX; and Marsh et al.—four cycles of mFFX pre- and postsurgery, are in progress) (clinicaltrials.gov). The latter approach is intriguing as early systemic treatment, prior to surgical intervention, is attractive for many reasons: better selection of patients for surgery based on the exclusion of those with rapidly progressive disease; better tumor exposure to chemotherapy prior to disruption of the vasculature; ability to gauge response; better tolerance of chemotherapy prior to debilitating surgery; and increased R0 resections. Furthermore, pancreatic cancer has been shown to be systemic from the earliest stages 42–44 and thus an early systemic approach is not only logical but may also be essential.

Previous studies of neoadjuvant therapy in resectable patients include gemcitabine plus radiation (73/86 were taken to surgery, with 64/86 undergoing successful surgery) 45; and gemcitabine plus nab-paclitaxel (14/25 completing the planned three cycles, with surgery in 20/25, 19/20 R0) 46; (9/16 undergoing surgery at the time of reporting, with 8/9 R0 resections) 47.

The University of Michigan reported improved 1- and 3-year OS, lower margin and node positivity, and minimal additional perioperative toxicity in a retrospective review of various neoadjuvant regimens in borderline resectable disease 48. University of Washington similarly reported almost doubling of OS in a small series of patients with both resectable and borderline resectable disease (neoadjuvant GTX vs. historical controls) 49, and Columbia was able to convert 57% of inoperable patients to operable with 49% R0 resections 50. Finally, the Medical College of Wisconsin reported on mFFX followed by radiation therapy in borderline resectable disease and found this approach both safe and favorable compared to historical controls 51. The ALLIANCE/Intergroup A021101 study is examining the feasibility of mFFX for four cycles followed by RT with oral capecitabine in a multi-institutional setting. Gemcitabine is given in the adjuvant space. The primary endpoint is 1-year OS and there are multiple levels of quality control to ensure validity (clinicaltrials.gov).

## How Best Can Response to FFX Therapy be Assessed?

Both serologic and radiographic response to therapy has come under increasing scrutiny. CA 19-9 has been used for decades as a serum marker in pancreatic cancer in Lewis antigen-positive individuals 52,53. However, this is complicated by the fact that biliary obstruction, pancreatitis, intestinal inflammation, and even elevated blood glucose 54 all lead to an increase in CA 19-9. While there is evidence that there is a difference in outcome between no responders and stable or good responders 55,56, there are opposing findings suggesting that there may be no correlation 57, and additional data are awaited.

Change in tumor dimensions, as assessed on CT scan and/or MRI, is both challenging to measure and often insignificant 58. In a study of 129 patients with borderline resectable tumors, post therapy, presurgical imaging suggested that only 1% had been down staged, 78% had no change, and 21% had progressive disease 59. In fact, 66% were able to undergo resection with 95% R0 resections. Provided the patient has acceptable performance status and no evidence of metastatic disease, even where there is no obvious radiographic response, surgery should proceed as pathology may indicate clear-cut treatment effect 60. Whether pathologic response has any meaning in the clinical context awaits further clarification, but initial reports suggest that more than 5% viable cells in the final specimen portends a bad outcome 61,62.

While endoscopic ultrasound can be valuable 63, novel ways of imaging the tumor, such as perfusion imaging 64, dynamic PET scans 65 and routine CT scan derived mass transport parameters, are increasingly being incorporated into investigational algorithms 66.

## How is FFX Optimally Combined with Radiation Therapy?

Many protocols in borderline and locally advanced, unresectable disease switch to radiation therapy following initial FFX 51. However, the precise role of radiation in these settings is the subject of ongoing debate. The LAP 07 study found that in locally advanced disease, chemo/radiation had no effect on OS compared to continued chemotherapy alone (over 40% of patients developed metastatic disease prior to being randomized to radiation or not) in those patients stable after an initial phase of gemcitabine ± erlotinib 67. Updated results in 2014 suggested less local recurrence in the CRT arm (34% vs. 65%, *P* < 0.0001). The true impact of radiation may not be fully evaluable until systemic disease control improves further. An upcoming study will re-explore this question: the three-arm randomized phase II RTOG 1201 study, which is evaluating systemic chemotherapy alone (gemcitabine plus nab-paclitaxel) versus capecitabine plus standard versus intensified local RT (50.4 Gy vs. 63 Gy) preceded and followed by systemic therapy. Given that FFX is notably more active than gemcitabine in metastatic disease 6, the combination of radiation with FFX deserves to be examined and novel approaches such as SBRT 68,69, may make it possible to do so.

## How Best to Combine FFX with Other Regimens?

Recently, the MPACT study reported on gemcitabine plus nab-paclitaxel (GN) versus gemcitabine in 861 patients with metastatic pancreatic cancer 56. Median OS was 8.5 versus 6.7 months (HR for death, 0.72; 95% CI = 0.62–0.83; *P* < 0.001), and progression-free survival was 5.5 versus 3.7 months (HR = 0.69, 95% CI = 0.58–0.82; *P* < 0.0001). While less than that of FFX in the PRODIGE 4/ACCORD 11 study—11.1 months 6, median OS is significant enough to be of major interest, raising the issue of how best to integrate these two regimens in a comprehensive treatment plan. One of the more interesting questions is whether there is synergism, and whether pretreatment with GN would alter the cancer-associated stroma such that FFX would be more effective. A recent phase II study used up to six cycles of GN followed by consolidation with FFX for up to 12 cycles and was deemed feasible 70. A case report from Germany, reported success with this approach in locally advanced disease 71.

The efficacy of GN following failure of FFX is unknown. In a retrospective study from Yale, 23 patients were so treated with an estimated time-to-treatment failure of 11 weeks, about half of that in first-line GN 72. Interestingly, dose densities of only 56.9% and 63.5% for nab-paclitaxel and gemcitabine, respectively, were achieved which suggest that alternative dosing schedules should be examined.

Innovative approaches currently under investigation include addition of a Hedgehog inhibitor to FFX 73; combination of FFX, SBRT, and GVAX as adjuvant therapy; and a combination of FFX and hyperacute vaccine in borderline and locally advanced disease. As we learn more, it is hoped that future study design will be based on biology and molecular profiling of tumors, rather than empiricism or intuition.

## How Do We Use Molecular Signatures in Planning FFX Treatment?

There is an increasing interest in the molecular profiling of cancers. Certainly, patients testing positive for a BRCA 1 or BRCA 2 mutation might have increased sensitivity to a platin 74, but this has uncertain practical value. PARP inhibitors might be more effective 75. From the Pancreatic Cancer Genome Project, we know that pancreatic cancers contain an average of 63 genetic alterations, the majority of which are point mutations 76. A core set of 12 cellular signaling pathways and processes are defined by these alterations in 67–100% of tumors. KRAS, Hedgehog, Wnt/Notch, SMAD4, and TGF-β signaling pathways are key, with abnormalities of one or more of these pathways in 100% of cancers. The effects on therapy with FFX are as yet unknown.

Candidates for future study include predictors of drug metabolism and toxicity—ERCC1 expression (oxaliplatin) 77, UGT 1A1 genotype (irinotecan) 28, thymidylate synthase expression (5FU) 78, HENT-1 expression (gemcitabine—both positive and negative studies) 79,80 and SPARC expression—both nab-paclitaxel 81 and gemcitabine 82.

## What Important Clinical Studies are Currently Underway in Pancreatic Cancer Using FFX Alone or in Combination?

As a final note, it is relevant to include a table of selected current and ongoing studies using FFX in all stages of pancreatic cancer (Table 3). These studies have been selected from many for their potentially significant impact on the use of this regimen in the future. It may once again be noted that FFX is very frequently modified.

**Table 3:** Selected current studies using FOLFIRINOX in all stages of pancreatic cancer

Setting	Study	Regimen	Goal	Opened
Resectable neoadjuvant	NorthShore/University of Chicago	mFFX—no 5FU bolus—four cycles pre- and postop	Assess safety and efficacy (R0, ORR, PFS, and OS)	August 2012
	Pilot study			
Resectable neoadjuvant	Indiana University	Standard full dose FFX—four cycles preoperatively	Assess safety and efficacy (Path CR, DFS, OS, ORR)	June 2014
	Phase II study			
Resectable neoadjuvant	Yale/NCI	mFFX—no 5FU bolus—six cycles pre and post op	Assess safety and efficacy (R0, path CR, PFS, and OS)	January 2014
	Phase II study			
Resectable adjuvant	PRODIGE 24/ACCORD 24	mFFX—no 5FU bolus—versus gemcit, each for 24 weeks	DFS, OS, specific survival	February 2012
	Phase III			
Resectable adjuvant	Krankenhaus Nordwest	Standard full dose FFX—six cycles pre and postop vs. gemcit postop	Assess safety and efficacy (OS, PFS, R0, path CR)	Opening pending
	Phase II/III			
Resectable adjuvant	Sidney Kimmel Comprehensive Cancer Center	SBRT plus Vaccine (GVAX)/cyclophosphamide then standard full dose FFX—six cycles with GVAX	Toxicity, safety, OS, DFS, TTF	April 2012
	Pilot Study			
Borderline resectable	ALLIANCE A021101	mFFX—no 5FU bolus—four cycles, then RT/cape gemcit postop	Accrual rate, toxicity, CR/PR, completion of all therapy, R0/R1	March 2013
	Pilot study			
Borderline resectable	Medical University of South Carolina	mFFX—no 5FU bolus—six cycles then RT/cape	R0/R1 resection (OS, TTR, ORR, path CR) and safety	August 2012
	Phase II			
Borderline resectable	University of Maryland	mFFX—no 5FU bolus—four cycles then SBRT	Resectability, DFS, OS, TTR, path CR and safety	September 2013
	Pilot Study			
Locally advanced	UNC LINEBERGER	Standard full dose FFX	Assess safety and efficacy (OS, PFS, ORR)	September 2012
	Phase II			
Locally advanced	Standard full dose FFX—four cycles then SBRT	OS, radiologic RR, Resection rate, PFS, Biologic predictive markers	July 2014
				Foundation for Liver Research/Erasmus Medical Center Phase II
Locally advanced	Massachusetts General Hospital/NCI	Standard full dose FFX—eight cycles plus losartan then proton beam RT	Feasibility, PFS, OS, toxicity, downstaging, gene mutations	March 2013
	Phase II			
Metastatic disease	University of Chicago	Modified FFX—irinotecan dose determined by UGT1A1 status; no 5FU bolus	DLT in course 1; RR, cumulative dose intensity of irinotecan	July 2012
	Phase II			
Metastatic disease	Institut Cancerologie de l'Ouest	Modified FFX—irinotecan dose determined by UGT1A1 status; 5FU dose by DPD expression	Safety, toxicity and efficacy (OS, PFS)	May 2014
	Phase II			
Metastatic disease	Centre Val d'Aurelle—Paul Lamarque	Standard Gemcitabine plus nab-paclitaxel followed by standard FFX	MTD; Phase II dosing; RR	August 2013
	Phase I–II			

ORR, overall response rate; PFS, progression-free survival; OS, overall survival; CR, complete remission; gemcit, gemcitabine; SBRT, stereotactic body radiation therapy; TTF, time-to-treatment failure; cape, capecitabine; TTR, time to response; DLT, dose-limiting toxicity; DPD, dihydropyrimidine dehydrogenase; MTD, maximum-tolerated dose; FFX, FOLFIRINOX; postop, postoperative.

## Summary

FFX has had a major impact on the treatment of pancreatic cancer. As experience with this regimen has accrued, and as we have learned how to manage the toxicities, we have been presented with a new set of questions: the effect of frequent modifications; optimal use in all stages of pancreatic cancer; integration with both established and emerging therapies; how to evaluate response; and the incorporation of evolving molecular data. Furthermore, while metastasectomy in pancreatic cancer has historically been fraught with futility and failure, the markedly improved activity of FFX 5,6 could mean that the time to study surgery plus FFX (in highly selected patients) is near 83,84. The next few years should prove to be exciting for all working to improve the outlook for this challenging group of patients.

## Conflict of Interest

None declared.
